# Microbial degradation of lignin: Role of lignin peroxidase, manganese peroxidase, and laccase

**Published:** 2004-05-01

**Authors:** Takayoshi Higuchi

**Affiliations:** Professor Emeritus of Kyoto University, Wood Research Institute, Kyoto University, Uji, Kyoto 611-0011

**Keywords:** Microbial degradation of lignin, lignin peroxidase (LiP), manganese peroxidase (MnP), laccase (LA), aromatic ring cleavage, side chain cleavage

## Abstract

Lignin peroxidase (LiP), laccase (LA) and manganese peroxidase (MnP) of white-rot basidiomycetes such as *Phanerochaete chrysosporium*, *Coliorus versicolor*, *Phlebia radiata* and *Pleurotus eryngii* catalyze oxidative degradation of lignin substructure model compounds and synthetic lignins (DHPs). Side chain- and aromatic ring cleavage products of both phenolic and non-phenolic substrates oxidized by LiP were isolated and characterized by NMR and MS. The cleavage mechanism was elucidated by using ^18^O, ^2^H, and ^13^C labeled lignin substructure dimers with ^18^O_2_ and H_2_^18^O. Recent studies suggested that LiP is capable of oxidizing lignin directly at the protein surface via a long-range electron transfer process. LA and MnP, which oxidize phenolic but not non-phenolic moieties, generally degrade lignin stepwise from phenolic moieties. However, recent studies indicated that MnP and LA can degrade both phenolic and non-phenolic aromatic moieties of lignin with some special mediators.

## Introduction

**Fig. 1:**
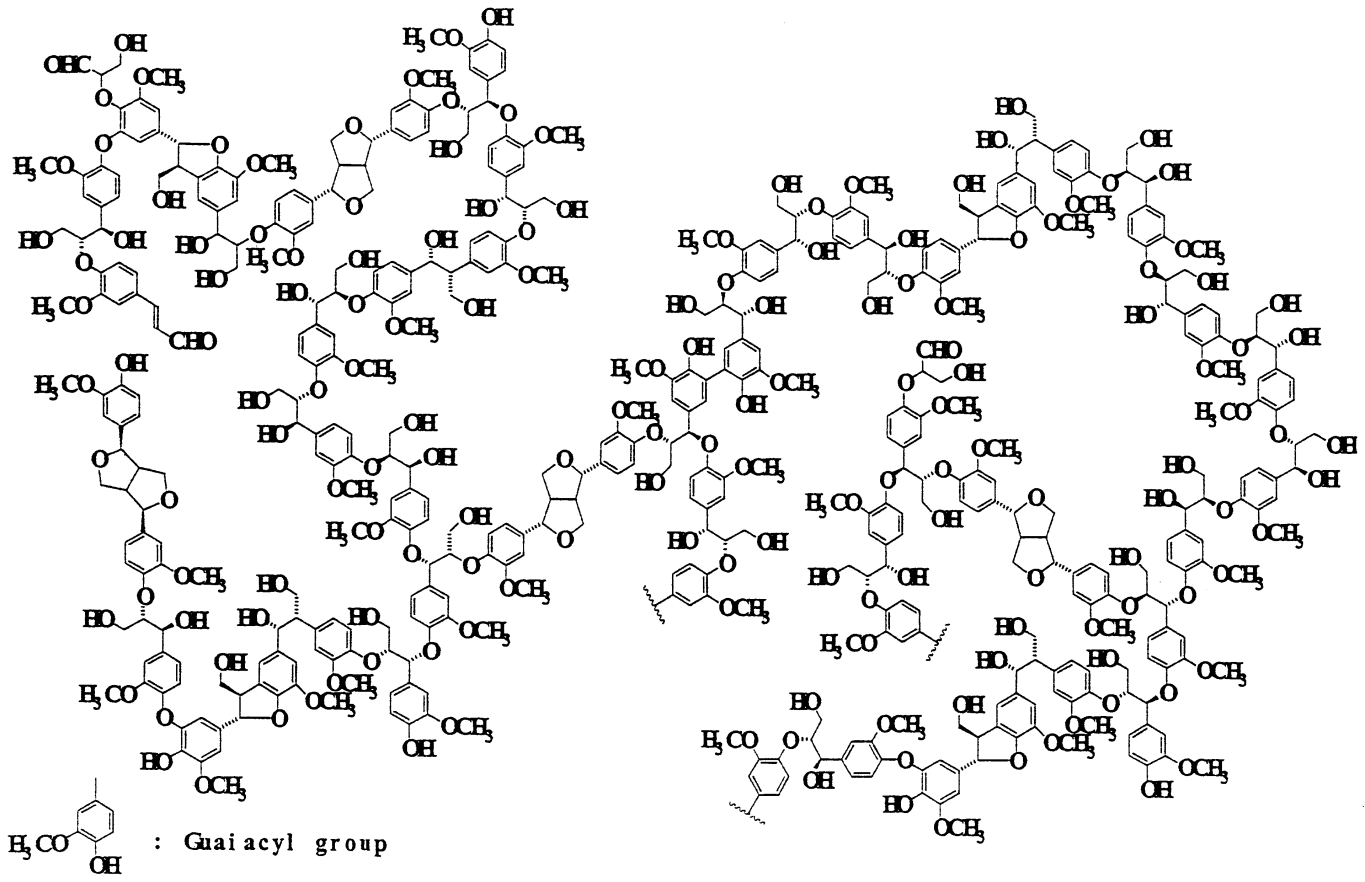
A structural model of softwood lignin (guaiacyl lignin).

Lignins are three dimentional phenylpropanoid polymers linked by several different carbon-to-carbon and ether linkages between phenylpropane units most of which are not readily hydrolysable. (Fig. 1)1) Lignin is considerably resistant to microbial degradation in comparison with polysaccharides and most other biopolymers.

Nevertheless, white-rot basidiomycetes such as *Coriolus versicolor*, *Phanerochaete chrysosporium*, *Phlebia radiata*, *Pleurotus eryngii* etc. are known as typical lignin-degrading microorganisms. During the past 20 years, knowledge of the chemistry and biochemistry of lignin biodegradation by white rot basidiomycetes progressed substantially mainly through two complementary approaches. 1) Chemical and spectrometric analyses of proto- vs. degraded lignins, and 2) biochemical elucidation of the degradation mechanism of lignin substructure model compounds and synthetic lignins (DHPs).2)–6)

Higuchi and Nakatsubo7) synthesized several oligolignols containing major lignin substructures such as ***β***-O-4, the most frequent interphenylpropane linkage (40–60% in lignin), ***β***-5 (10%), ***β***-1 (5%), and ***β***-***β*** (<5%) linkages. The lignin substructure oligomers were used to elucidate lignin degradation mechanisms by *P. chrysosporium* and *C. versicolor*, and their enzymes, LiP and laccase (LA).

## Mechanism of side chain cleavage of lignin substructure model compounds by lignin peroxidase (LiP)

### 1. β-1 Compounds

Kirk and Nakatsubo8) found for the first time that a deuterated non-phenolic 1,2-diarylpropane-1,3-diol model oligomer is degraded via C***α***-C***β*** cleavage by a ligninolytic culture of *P. chrysosporium* to give phenylglycol, ***α***-hydroxyacetophenone and benzaldehyde products, with retention of hydrogen atoms at C***α*** and C***β***. They further found using ligninolytic culture experiments with ^18^O that the benzyl hydroxyl oxygen atom of the phenylglycol was derived from molecular oxygen.

Subsequently Tien and Kirk9) and Glenn *et al*.10) discovered the enzyme lignin peroxidase (LiP) which catalyzes C***α***-C***β*** cleavage in the propyl side chains of ***β***-1 compounds, in agreement with *in vivo* experiments.

**Fig. 2:**
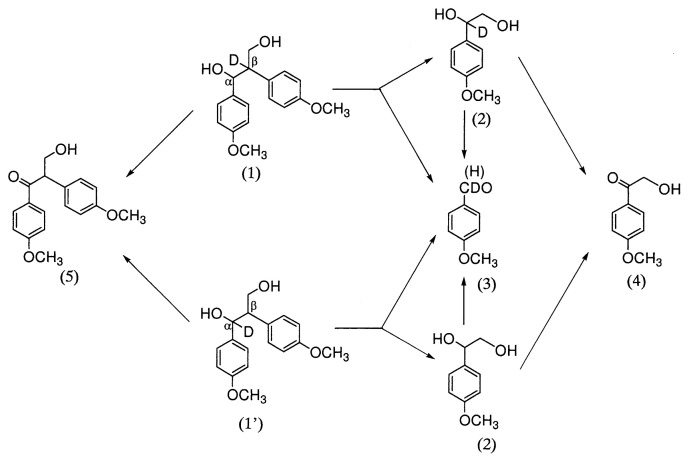
Degradation pathways of deutrated ***β***-1 lignin models (1,1’) by lignin peroxidase (LiP) of *P. chrysosporium*. (D), deuterium; (2), 4-methoxyphenylglycol; (3), 4-methoxybenzaldehyde; (4), 1-(4-methoxyphenyl)-2-hydroxyethanone; (5), 1-(4-methoxyphenyl)-2-(4-methoxyphenyl) propane-1-one-3-ol.

Habe *et al*.11) synthesized deuterated non-phenolic 1,2-diarylpropane-1,3-diols as substrates for experiments with LiP of *P. chrysosporium*, and found the formation of phenylglycol, ***α***-hydroxyacetophenone and benzaldehyde products with retention of the deuterium at C***α*** and C***β*** of the side chain. The result confirmed that hydrogen abstraction is not involved in the C***α***-C***β*** bond cleavage (Fig. 2).

Kersten *et al*.12) and Hammel *et al*.13),14) showed that LiP acts by catalyzing the 1-electron oxidation of aromatic rings, forming cation radicals, which undergo a variety of nonenzymatic reactions.

### 2. β-O-4 Compounds

**Fig. 3:**
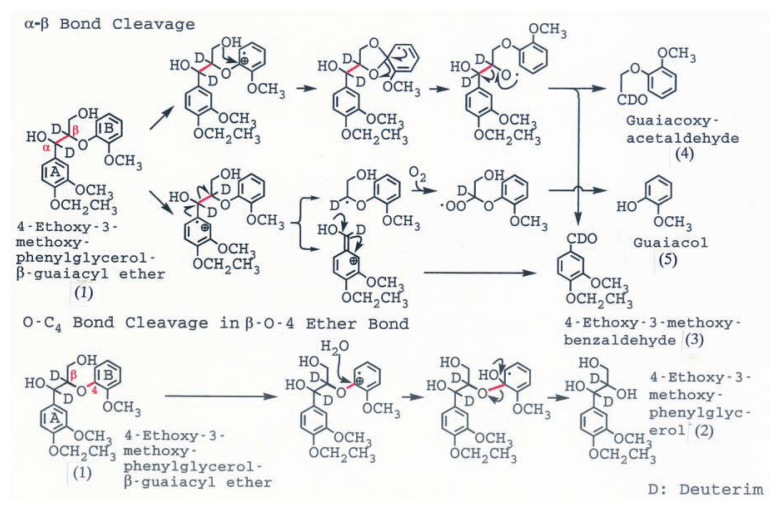
Degradation of deuterated arylglycerol-***β***-aryl ether lignin substructure models by LiP of *P. chrysosporium*. (1), ***α***,***β***-dideuterated 4-ethoxy-3-methoxyphenylglycerol-***β***-guaiacyl ether; (2), 4-ethoxy-3-methoxyphenylglycerol; (3), 4-ethoxy-3-methoxybenzaldehyde; (4), guaiacoxyacetaldehyde; (5), guaiacol.

Umezawa *et al*.4),15) found that ***α***,***β***-dideuterated 4-ethoxy-3-methoxyphenylglycerol-***β***-guaiacyl ether (Fig. 3, (1) was converted by LiP to 4-ethoxy-3-methoxybenzaldehyde (3), and guaiacoxyacetoaldehyde (4) and guaiacol (5) by C***α***-C***β*** cleavage, and subsequent O-C***β*** cleavage, and 4-ethoxy-3-methoxyphenylglycerol (2) by O-C_4_ cleavage. Mass spectrometric analysis showed that deuterium at C***α*** and C***β*** of the 4-ethoxy-3-methoxyphenylglycerol, of 4-ethoxy-3-methoxybenzaldehyde and of guaiacoxyacetaldehyde were almost quantitatively retained after the C***α***-C***β*** and O-C_4_ bond cleavages. (Fig. 3) The results clearly showed that C***α***-C***β*** cleavage and O-C_4_ cleavage occurred via the cation radical intermediates by one electron oxidation of the aromatic ring of the substrate by LiP.

In further investigation4) we identified an alternative C***α***-C***β*** cleavage reaction of a ***β***-O-4 model compound, 4-ethoxy-3-methoxyphenylglycerol-***β***-^18^O-guaiacyl ether, to give 2-guaiacoxyethanol and benzyl alcohol probably via benzaldehyde, in ligninolytic cultures of *P. chrysosporium*. GC-MS analyses of the isolated products showed that ^18^O of the ether oxygen of the substrate was not retained in the 2-guaiacoxyethanol product. When 4-ethoxy-3-methoxyphenylglycerol(***γ***-^13^C)-***β***-guaiacyl ether used as substrate, the 2-guaiacoxyethanol product was labeled with ^13^C at the 2-position but not the 1-position.

## Mechanism of aromatic ring cleavage of lignin substructure model compounds by LiP

Kirk and Chang16) compared white-rotted lignin polymer (isolated and purified from white-rotted wood) with non-degraded lignin, using a variety of chemical and physical methods. Among their conclusions was that aromatic rings had been cleaved while still in the polymer.

**Fig. 4:**
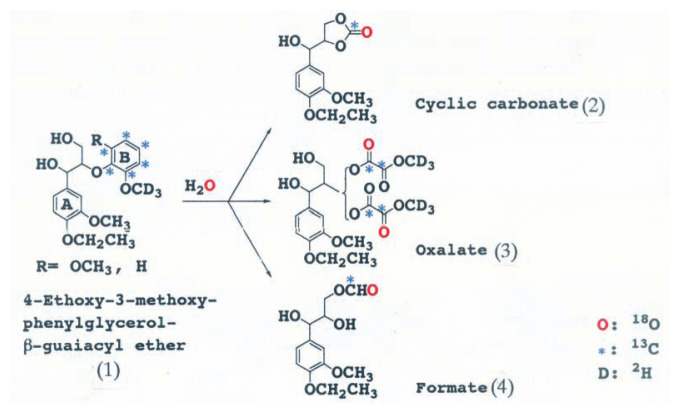
Aromatic ring cleavage products of arylglycerol-***β***-aryl-U-^13^C, OCD_3_ ethers (1) by LiP of *P. chrysosporium*. (2), 4-ethoxy-3-methoxyphenylglycerol-***β***,***γ***-cyclic carbonate; (3), 4-ethoxy-3-methoxyphenylglycerol-***β***-methyl oxalate; (4), 4-ethoxy-3-methoxyphenylglycerol-***γ***-formate.

The mechanism of aromatic ring cleavage of lignin by fungi, however, remained unsolved until 1985. Umezawa and Higuchi3)–5),15) synthesized 4-ethoxy-3-methoxyphenylglycerol-***β***-guaiacyl[U-ring^13^C,OCD_3_] ether, and 4-ethoxy-3-methoxyphenylglycerol-***β***-syringyl[U-ring^13^C,OCD_3_] ether as substrate to elucidate the mechanism of aromatic ring cleavage of the model compounds. The compounds were incubated with ligninolytic cultures of *P. chrysosporium* in the presence of H_2_^18^O. We isolated and identified for the first time ***β***, ***γ***- and ***α***,***β***-cyclic carbonates, formate and oxalate esters of arylglycerol from the reaction mixtures as aromatic ring cleavage products (Fig. 4).15) We finally identified a muconate ester of arylglycerol as an initial ring cleavage product of the dimers by LiP.6)

**Fig. 5:**
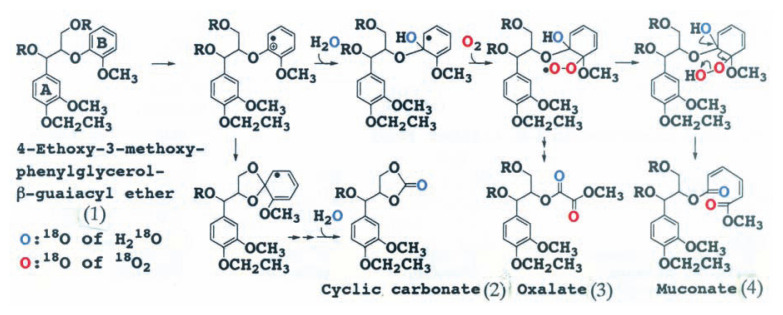
Mechanism of aromatic ring cleavage of ***β***-O-4 lignin substructure models by LiP of *P. chrysosporium*. (1), 4-ethoxy-3-methoxyphenylglycerol-***β***-guaiacyl ether; (2), 4-ethoxy-3-methoxyphenylglycerol-***β***, ***γ***-cyclic carbonate; (3), 4-ethoxy-3-methoxyphenylglycerol-***β***-methyl oxalate; (4), 4-ethoxy-3-methoxyphenylglycerol-***β***-mucorate.

The cleavage mechanism of the aromatic ring was further elucidated by experiments using ^2^H, ^13^C and ^18^O labeled dimers with ^18^O_2_ and H_2_^18^O. The results showed that the mechanism of aromatic ring cleavage of lignin is completely different from the aromatic ring cleavage reaction for catechol derivatives by dioxygenases: LiP catalyzes the one electron oxidation of the aromatic ring (B) of arylglycerol-***β***-aryl ether to give aromatic cation radicals which are attacked by H_2_O, and that the resulting radicals couple with dioxygen to afford the muconate ester of arylglycerol (Fig. 5).

## Cleavage of side chains and aromatic rings of a synthetic lignin (DHP) by LiP

Umezawa and Higuchi4),17) found that most of the initial stage of degradation reaction of ***β***-O-4 lignin substructure model dimers was catalyzed by LiP. A synthetic lignin (DHP: dehydrogenation polymer of coniferyl alcohol prepared using horseradish peroxidase, M. W. >2200) was prepared and subjected to degradation with LiP to elucidate the mechanism of lignin degradation by this enzyme.18)

As the case of the degradation of ***β***-O-4 lignin substructure model dimers by LiP, the cyclic carbonates and formate ester of arylglycerols, and arylglycerol were isolated from degradation products of the DHP by LiP ; the chemical structures of the products were identified by GC-MS. These results indicated that the lignin polymer is really degraded by the LiP of white-rot fungi.

## Active sites of LiP to substrates

Doyle and his group19) recently found that Trp 171 of LiP protein is hydroxylated at the C***β*** position. They found that the hydroxylation process in both wild type and recombinant LiP isozyme H_8_ is autocatalytic and that Trp 171 may be implicated in catalysis. Site directed mutagenesis of recombinant enzymes with Trp 171 substituted by Phe (W171F) or Ser (W171S) lost all activity for veratryl alcohol (VA; a LiP substrate) but not for two dye substrates. The result suggested two distinct substrate interaction sites in LiP, a heme-edge site, and a novel site centered around Trp 171 which is required for the oxidation of VA. Stop-flow kinetic studies strongly suggested that an electron-transfer pathway exists within the enzyme protein leading from the heme to a surface site in close proximity to Trp 171.

Johjima *et al*.20) confirmed that the binding site of LiP for VA is Trp 171 by using three different chemically modified LiPs against VA acting as a reducing substrate, a reducing reagent for the rapid conversion of LiPIII back to native LiP, and as an enzyme-bound redox mediator. They21) further studied the binding properties of LiP for synthetic lignin (DHP) by resonant mirror biosensor techniques, and found that among several ligninolytic enzymes only LiP specifically binds to DHP. Kinetic analysis showed that the binding is reversible, and LiP is capable of oxidizing lignin directly at the protein surface by a long-range electron transfer process. A close look at the crystal structure suggested that LiP possesses His-239 as a possible lignin-binding site on the surface, which is linked to Asp-238. This Asp residue is hydrogen-bonded to the proximal His-176. The His-Asp proximal-His motif would be a possible electron transfer route to oxidize polymeric lignin.

Tien’s group22) studied on the active site of LiP with respect to substrate size using either fungal or recombinant wild type, as well as mutated, recombinant LiPs. A nonphenolic tetrameric lignin model that contains ***β***-O-4 linkages was used as substrate. Both natural and recombinant LiPs oxidized the tetrameric model forming four products, tetrameric, trimeric, dimeric, and monomeric carbonyl compounds. The result indicated that LiP is able to attack any of C***α***-C***β*** linkages in the tetrameric compound and that the substrate-binding sites is thus well exposed. Mutation of a Trp residue (W171S) completely inhibited the oxidation of the tetramer model. These results are consistent with LiP having an exposed active site capable of directly interacting with the lignin polymer without the need for low molecular weight mediators, such as VA.

## Manganese peroxidase (MnP)

Following the discovery of LiP in *P. chrysosporium*,9),10) manganese peroxidase (MnP) secreted from the same fungus was found as another lignin degrading enzyme by Gold’s group,23),24) and Crawford’s group,25) respectively, and subsequent investigations have shown that MnP is distributed in almost all white-rot fungi.35)

Ten extracellular peroxidase isozymes were purified from the culture of *P. chrysosporium*.26) These enzymes were designated H_1_ to H_10_, according to their order of elution from an anion exchange column. Isoenzymes H_1_, H_2_, H_6_, H_7_, H_8_, and H_10_ were identified as LiP isoenzymes, and H_3_, H_4_, H_5_, and H_9_ as MnP isoenzymes, respectively.

MnP oxidizes phenolic compounds as well as Mn^2+^ to Mn^3+^. Mn^3+^ is stabilized by fungal chelators such as oxalic acid, and the chelated Mn^3+^ oxidizes phenolic compounds. MnP, in the presence of sodium malonate, Mn^2+^ and H_2_O_2_, was found to catalyze C***α***-C***β*** cleavage, C***α***-oxidation and alkyl-aryl cleavages of phenolic ***β***-1 and ***β***-O-4 lignin substructures.27),28)

It has been proposed that chelated Mn^3+^ acts as low-molecular weight, diffusible redox-mediator that attacks the phenolic lignin structure. Further investigations 29)–31) showed that the chelated Mn^3+^ system generates reactive intermediates (peroxy radicals) from unsaturated fatty acids such as linoleic acid and their derivatives (lipids).

The MnP-lipid system is strong enough to degrade C***α***-C***β*** and ***β***-aryl ether bonds in not only phenolic but also nonphenolic lignin model dimmers.

Hammel and his group32) found that wood block cultures and defined liquid medium cultures of *Ceriporiopsis subvermispora* rapidly depolymerized and mineralized a ^14^C-labeled, polyethylene glycol-linked high molecular weight ***β***-O-4 lignin model compound that represents major nonphenolic structure of lignin. The fungus cleaved the model between C***α*** and C***β*** to release benzylic fragments. The fungal degradation on the model and methylated lignin was significantly faster in the presence of Tween 80, a source of unsaturated fatty acids.

Wariishi *et al*.33) also found that MnP catalyzes substantial depolymerization of DHP by purified MnP of *P. chrysosporium* in the presence of malonic acid as the chelator. Both guaiacyl- and guaiacyl-syringyl lignin models were degraded substantially.

However, identification of cleavage products of side chain and aromatic ring of lignin substructure models and DHP by MnP, and the chemical degradation mechanism have scarcely been investigated.

## Versatile peroxidase (VP)

Versatile peroxidases (VP) that can oxidize Mn^2+^ as well as phenolic and non-phenolic aromatic compounds have been isolated from *Pleurotus* and *Bjerkandera*.34) VP oxidizes Mn^2+^ to Mn^3+^, degrades the nonphenolic lignin model veratrylglycerol-***β***-guaiacyl ether yielding veratryl aldehyde, and oxidizes veratryl alcohol and p-dimethoxybenzene to veratryl aldehyde and p-benzoquinone respectively as LiP does. A review on lignin conversion by MnP has been published recently.35)

## Laccase (LA)

In 1928 Bavendamm36) discovered by cultivating wood-rotting fungi in an agar medium containing several phenolic compounds, such as gallic acid, tannic acid, and hydroquinone, that white-rot fungi produced a large darkened zone around the mycerial mat, but no zone of darkening was associated with the growth of brown-rot fungi. Davidson *et al*.37) subsequently investigated the reaction using 210 species of wood-rotting fungi, and concluded that the white-rotting type coincides with Bavendamm’s reaction in general, and that the reaction is helpful in identifying fungi. The enzyme responsible for Bavendamm’s reaction was extensively studied in the next 10 years, and characterized to be laccase (LA).38) LA, p-diphenol oxidase (EC 1.10.3.2) has been isolated and characterized as a blue, copper containing oxidase from a lac tree (Rhus spp) and several fungi. White rot fungi constitutively produce laccase during primary metabolism.39)

### 1. Degradation of β-1 model compounds

**Fig. 6:**
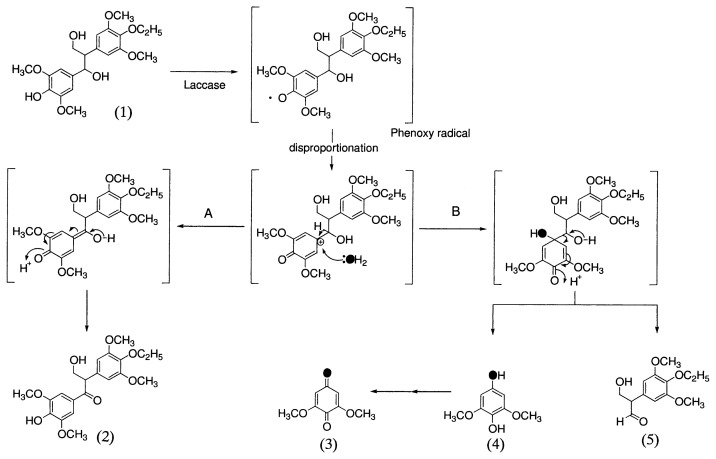
Mechanisms of C***α*** oxidation (A-route), C***α***-C***β***-cleavage and alkyl-phenyl cleavage (B-route) of phenolic ***β***-1 lignin substructure model compounds by laccase (LA) of *C. versicolor*. (1), 1-(3,5-dimethoxy-4-hydroxyphenyl)-2-(3,5-dimethoxy-4-ethoxyphenyl)propane-1,-3-diol; (2), 1-(3,5-dimethoxy-4-hydroxyphenyl)-2-(3,5-dimethoxy-4-ethoxyphenyl)-3-hydroxypropanone; (3), 2,6-dimethoxy-p-benzoquinone; (4), 2,6-dimethoxy-p-hydroquinone; (5), 2-(3,5-dimethoxy-4-ethoxyphenyl)-3-hydroxypropanal, (●), ^18^O.

Kawai *et al*.40) found that phenolic ***β***-1 model compounds are degraded by LiP of *P. chrysosporium* and LA of *C. versicolor* via similar pathways. 1-(3,5-Dimethoxy-4-hydroxyphenyl)-2-(3,5-dimethoxy-4-ethoxyphenyl)-propane-1,3-diol (1, Fig. 6) was converted by LA of *C. versicolor* to 1-(3,5-dimethoxy-4-hydroxyphenyl)-2-(3,5-dimethoxy-4-ethoxyphenyl)-3-hydroxypropanone (2)by C***α*** oxidation, 2-(3,5-dimethoxy-4-ethoxyphenyl)-3-hydroxypropanal (5), 2,6-dimethoxy-p-hydroquinone (4) and its benzoquinone (3) by alkyl-phenyl cleavage (Fig. 6). Their experiment further showed that ^18^O of ^18^O_2_ was incorporated into etahnone, and _18_O of H_2_^18^O into hydroquinone and benzoquinone, respectively.

Based on the structures of the degradation products and the isotopic experiments they concluded that three types of reactions proceeded via phenoxy radicals of the substrates generated by LA; 1) C***α***-C***β*** cleavage between C_1_ and C_2_, 2) alkyl-phenyl cleavage between C_1_ and aryl group, and 3) C***α*** oxidation.

Recently yellow laccase as well as blue laccase have been isolated from solid-state and submerged culture of *Panus tigrinus*. The yellow laccase had no blue maxima in the absorption spectrum, but catalyzed oxidation of VA and a non-phenolic ***β***-1 dimer. The yellow laccase was suggested to be formed as a result of blue laccase modification by products of lignin degradation, which might play a role as natural electron-transfer mediators for the oxidation of nonphenolic substances. 41)

### 2. Degradation of β-O-4 model compounds

Kirk *et al*.42) worked on degradation of the lignin model compound syringylglycerol-***β***-guaiacyl ether by *Polyporus versicolor* and *Stereum frustulatum*. They found that the benzyl alcohol group of the substrate was oxidized to a carbonyl group, giving ***α***-guaiacoxyacetosyringone by whole culture of *S. frustulatum* and the culture filtrate of *P. versicolor*. The alkylphenyl carbon-to-carbon bond in both syringylglycerol-***β***-guaiacyl ether and ***α***-guaiacoxyacetosyringone was cleaved by culture filtrate of *P. versicolor* with formation of guaiacoxyacetaldehyde and guaiacoxyacetic acid, respectively. The syringyl moieties of both parent compounds were converted to 2,6-dimethoxy-p-benzoquinone by culture filtrate of. *P. versicolor*. Laccase also effected all the above reactions.

Kawai *et al*.43) recently investigated the degradation of syringylglycerol-***β***-guaiacyl ether by LA of *C. versicolor*. They showed that the substrate is mainly converted to the ***α***-carbonyl dimer, 2,6-dimethoxyhydroquinone, and glyceraldehyde 2-guaiacyl ether by alkyl-phenyl cleavage, and to guaiacol by O-C***β*** cleavage. Syringaldehyde and guaiacoxyethanol as direct C***α***-C***β*** cleavage products of the substrate were not found.

**Fig. 7:**
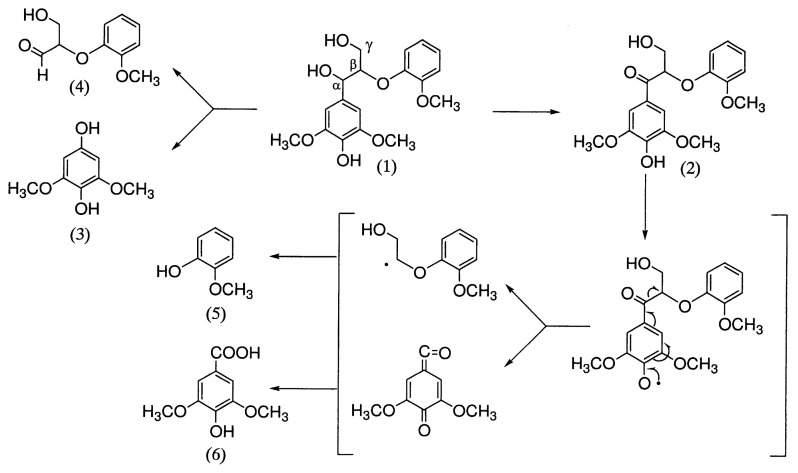
Mechanism of side chain cleavage of a phenolic ***β***-O-4 lignin substructure model by LA of *C. versicolor*. (1), syringylglycerol-***β***-guaiacyl ether; (2), ***α***-carbonyl dimer; (3), 2,6-dimethoxyhydroquinone; (4), glyceraldehyde-2-guaiacyl ether; (5), guaiacol; (6), syringic acid.

Subsequent investigation to identify the pathway to give guaiacol showed that ***α***-carbonyl dimmer used as substrate is cleaved between C***α*** and C***β*** to give syringic acid and guaiacol as shown in Fig. 7. The result indicated that phenolic ***β***-O-4 compound is degraded not only by alkyl-phenyl cleavage, which has been proposed as a major LA-mediated degradative reaction, but also by C***α***-C***β***-cleavage of the C***α***-carbonyl dimmer previously formed by C***α*** oxidation by LA. The side chain cleavage of phenolic ***β***-O-4 lignin substructure model compounds with LiP and LA suggested that the same chemical principle, phenoxy radical as intermediate, is involved in the degradation of phenolic lignin substructure model compounds by both enzymes.

Recently, the degradation of nonphenolic lignin model compounds by LA in the presence of appropriate mediators such as 1-hydroxybenzotriazole (1-HBT) and 2,2’-azobis(3-ethylbenzthiazoline-6-sulfonic acid) has been reported.44) Kawai *et al*.45) found that LA of *C. versicolor* catalyzed C***α***-C***β*** cleavage, C***α***-oxidation, ***β***-ether cleavage, and aromatic ring cleavage of the non-phenolic ***β***-O-4 lignin model dimmer 1,3-dihydroxy-2-(2,6-dimethoxyphenoxy)-1-(4-ethoxy-3-methoxyphenyl)-propane in the presence of 1-HBT. They also found that the oxidation of the substrate in H_2_^18^O resulted in the incorporation of ^18^O into three aromatic ring cleavage products and a ***β***-ether cleavage product which are identical with those obtained by LiP.

### 3. Syringyl polymer

Syringyl lignin model polymer (MW>2200) was degraded by LA of *C. versicolor*.46) The polymer was depolymerized partially to form 2,6-dimethoxy-p-hydroquinone, 2,6-dimethoxy-p-benzoquinone, and syringaldehyde. NMR spectra of the degraded substrate suggested that the LA catalyzed the oxidation of benzylic hydroxyl groups to ketones at the polymer stage.

### 4. Aromatic ring cleavage

**Fig. 8:**
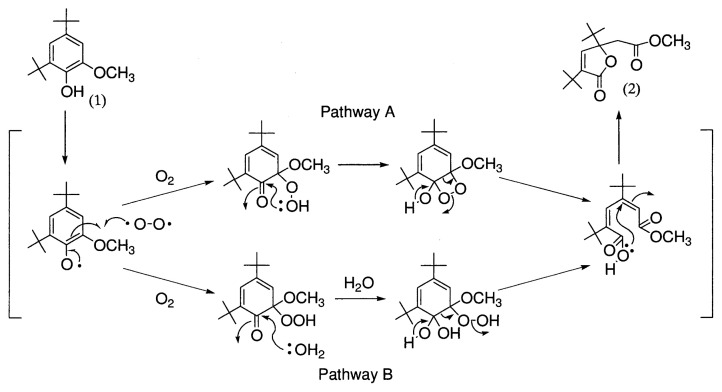
Mechanisms for degradation of 4,6-di-*t*-butylguaiacol by LA of *C. versicolor*. (1), 4,6-di-*t*-butylguaiacol; (2), muconolactone derivative.

Kawai *et al*.47) found that 4,6-di-*t*-butylguaiacol is converted by LA of *C. versicolor* to a ring cleavage product, the muconolactone derivative, which was previously identified by Gierer and Imsgard48) as a product in alkaline-oxygen oxidation of the same substrate. The experiment showed that ^18^O from ^18^O_2_ but not from H_2_^18^O is incorporated into the muconolactone derivative. Thus, the pathway A in Fig. 8 was proposed for ring cleavage of 4,6-di-*t*-butylguaiacol by LA.

## Conclusion

The main cleavage mechanisms of side chains and aromatic rings of lignin model compounds and synthetic lignin (DHP) by white-rot fungi and their enzymes LiP, and LA have been elucidated using ^2^H, ^13^C and ^18^O-labeled lignin substructure dimmers with ^18^O_2_ and H_2_^18^O. Side chain and aromatic rings of these substrates were cleaved via aryl cation radical and phenoxy radical intermediates in reactions mediated by LiP/H_2_O_2_, and laccase/O_2_/mediator.

Hydrogen peroxide is only required for the conversion of native LiP and MnP into two electron-deficient reactive species (compound I). Compound I of LiP abstracts stepwise two electrons from the aromatic ring of lignin substrate to yield aryl cation radicals or aryl cations, which are attacked by O_2_ or nucleophiles such as H_2_O and R-OH, respectively. The subsequent reactions of the cation radicals and cations are not controlled by the enzyme just as in the non-enzyme-directed coupling of phenoxy radicals of monolignol in lignin biosynthesis. Thus, the role of LiP, LA, and probably MnP in lignin biodegradation could be explained by the following unifying view.

### Enzymatic reaction

1. LiP/ H_2_O_2_ → Phenoxy radicals of phenolic units, and aryl cation radicals or cation radicals of non-phenolic units
2. LA/O_2_ → Phenoxy radicals of phenolic units
3. LA/O_2_+Mediators → Phenoxy radicals of phenolic units, and aryl cation radicals or cation radicals of nonphenolic units
4. MnP/H_2_O_2_+Mn^2+^ → Phenoxy radicals of phenolic units
5. MnP/H_2_O_2_+Mn^2+^+Mediators → Phenoxy radicals of phenolic units and aryl cation radicals or cation radicals of non-phenolic units

### Non-enzymatic reaction

1. Homolytic or heterolytic cleavage of side chains (C***α***-C***β***, alkyl-phenyl), and aromatic rings
2. O_2_ attack on carbon-centered radical intermediates
3. Nucleophilic attack on aryl cations and C***α*** cations by H_2_O and R-OH → Degradation products

Recent molecular investigations49) on ligninolytic enzymes have shown that *P. chrysosporium* has two gene families including ten LiP-type and three MnP-type genes coding different isoenzymes expressed during secondary metabolism. Many ligninolytic peroxidase genes from other white-rot fungi, and two VP genes from *Pleurotus eryngii* have been cloned.

Biochemical and biotechnological approaches to lignin biodegradation open up a new field in biomass conversion, such as biopulping50)–53) biobleaching, and treatment of Kraft bleaching effluents and related pollutants by lignin degrading basidiomycetes and their enzymes.54) A review article49) is referred to for molecular biology and engineering of lignin biodegradation.

## References

[1] Higuchi T. (2003) Pathways for monolignol biosynthesis via metabolic grids: coniferyl aldehyde 5-hydroxylase, a possible key enzyme in angiosperm syringyl lignin biosynthesis. Proc. Jpn. Acad., Ser. B 79, 227–236.

[2] Kirk T. K. Farrell R. L. (1987) Enzymatic “combustion”: the microbial degradation of lignin. Annu. Rev. Microbiol. 41, 465–505. 3318677 10.1146/annurev.mi.41.100187.002341

[3] Higuchi T. (1990) Lignin biochemistry: Biosynthesis and biodegradation. Wood Sci. Technol. 24, 23–63.

[4] Umezawa T. Higuchi T. (1991) Chemistry of lignin degradation by lignin peroxidases. In Enzymes in Biomass Conversion (eds. Leatham G. F. Himmel M. E.). ACS Symposium Series 460, American Chemical Society, Washington DC, pp. 236–246.

[5] Higuchi T. (1993) Biodegradation mechanism of lignin by white-rot basidiomycetes. J. Biotechnol. 30, 1–8.

[6] Umezawa T. Higuchi T. (1987) Formation of a muconate in aromatic ring cleavage of a ***β***-O-4 lignin substructure model by lignin peroxidase. Agric. Biol. Chem. 51, 2282–2284.

[7] Higuchi T. Nakatsubo F. (1980) Synthesis and biodegradation of oligolignols. Kemia-Kemi 9, 481–488.

[8] Kirk T. K. Nakatsubo F. (1983) Chemical mechanism of an important cleavage reaction in the fungal degradation of lignin. Biochem. Biophys. Acta 756, 376–384.

[9] Tien M. Kirk T. K. (1983) Lignin-degrading enzyme from the hymenomycete *Phanerochaete chrysosporium* Burds. Science 211, 661–663. 10.1126/science.221.4611.661 17787736

[10] Glenn J. K. Morgan M. A. Mayfield M. B. Kuwahara M. Gold M. H. (1983) An extracellular H_2_O_2_-requiring enzyme preparation involved in lignin biodegradation by the whiterot basidiomycete *Phanerochaete chrysosporium*. Biochem. Biophys. Res. Commun. 114, 1077–1083. 6615503 10.1016/0006-291x(83)90672-1

[11] Habe T. Shimada M. Umezawa T. Higuchi T. (1985) Evidence for deuterium retention in the products after enzymatic C-C and ether bond cleavages of deuterated lignin model compounds Agric. Biol. Chem. 49, 3505–3510.

[12] Kersten P. J. Tien M. Kalyanaraman B. Kirk T. K. (1985) The ligninase of *Phanerochaete chrysosporium* generates cation radicals from methoxybenzenes.J. Biol. Chem. 260, 2609–2612. 2982828

[13] Hammel K. E. Kalyanaraman B. Kirk T. K. (1986) Substrate free radicals are intermediates in ligninase catalysis. Proc. Natl. Acad. Sci. USA 83, 3708–3712. 3012530 10.1073/pnas.83.11.3708 PMC323592

[14] Hammel K. E. Tien M. Kalyanaraman B. Kirk T. K. (1985) Mechanism of oxidative C***α***-C***β*** cleavage of a lignin model dimmer by *Phanerochaete chrysosporium* ligninase: Stoichiometry and involvement of free radicals. J. Biol. Chem. 260, 8348–8353. 2989288

[15] Umezawa T. Nakatsubo F. Higuchi T. (1983) Degradation pathway of arylglycerol-***β***-aryl ethers by *Phanerochaete chrysosporium*. Agric. Biol. Chem. 47, 2677–2681.

[16] Kirk T. K. Chang H.-m. (1975) Decomposition of lignin by white-rot fungi. II. Characterization of heavily degraded lignins from decayed spruce wood. Holzforschung 29, 56–64.

[17] Umezawa T. Higuchi T. (1985) Aromatic ring cleavage in degradation of ***β***-O-4 lignin substructure by *Phanerochaete chrysosporium*. FEBS Lett. 182, 257–259.

[18] Umezawa T. Higuchi T. (1989) Cleavages of aromatic ring and ***β***-O-4 bond of synthetic lignin (DHP) by lignin peroxidase. FEBS Lett. 242, 325–329. 2914615 10.1016/0014-5793(89)80494-6

[19] Doyle W. A. Blodig W. Veith N. C. Piontek K. Smith A. T. (1998) Two substrate interaction sites in lignin peroxidase revealed by site-directed mutagenesis. Biochemistry 37, 15097–15105. 9790672 10.1021/bi981633h

[20] Johjima T. Wariishi H. Tanaka H. (2002) Veratryl alcohol binding sites of lignin peroxidase from *Phanerochaete chrysosporium*. J. Molecular Catalysis B: Enzymatic 17, 49–57.

[21] Johjima T. Itoh N. Kabuto M. Tokimura F. Nakagawa T. Wariishi H. Tanaka H. (1999) Direct interaction of lignin and lignin peroxidase from *Phanerochaete chrysosporium*. Proc. Natl. Acad. Sci. USA 96, 1989–1994. 10051582 10.1073/pnas.96.5.1989 PMC26724

[22] Mester T. Ambert-Balay K. Ciofi-Baffoni S. Banci L. Jones A. D. Tien M. (2001) Oxidation of a tetrameric nonphenolic lignin model compound by lignin peroxidase. J. Biol. Chem. 276, 22985–22990. 11304528 10.1074/jbc.M010739200

[23] Kuwahara M. Glenn J. K. Morga M. A. Gold M. H. (1984) Separation and characterization of two extracellular H_2_O_2_-dependent oxidases from ligninolytic cultures of *Phanerochaete chrysosporium*. FEBS Lett. 169, 247–250.

[24] Glenn J. K. Gold M. H. (1985) Purification and characterization of an extracellular Mn(II)-dependent peroxidase from the lignin degrading basidiomycete, *Phanerochaete chrysosporium*. Arch. Biochem. Biophys. 242, 329–341. 4062285 10.1016/0003-9861(85)90217-6

[25] Paszcynski A. Huynh V.-B. Crawford R. (1985) Enzymatic activities of an extracellular, manganese-dependent peroxidase from *Phanerochaete chrysosporium*. FEMS Microbiol. Lett. 29, 37–41.

[26] Cai D. Tien M. (1993) Lignin-degrading peroxidases of *Phanerochaete chrysosporium*. J. Biotechnol. 30, 79–90. 7763834 10.1016/0168-1656(93)90029-m

[27] Wariishi H. Valli K. Gold M. H. (1989) Oxidative cleavage of a phenolic diarylpropane lignin model dimmer by manganese peroxidase from *Phanerochaete chrysosporium*. Biochemistry 28, 6017–6023.

[28] Tuor U. Wariishi H. Schoemaker H. E. Gold M. H. (1992) Oxidation of phenolic arylglycerol-***β***-aryl ether lignin model compounds by manganese peroxidase from *Phanerochaete chrysosporium*: oxidative cleavage of an ***α***-carbonyl model compound. Biochemistry 31, 4986–4995. 1599925 10.1021/bi00136a011

[29] Bao W. Fukushima Y. Jensen K. A. Moen M. A. Hammel K. E. (1994) Oxidative degradation of non-phenolic lignin during lipid peroxidation by fungal manganese peroxidase. FEBS Lett. 354, 297–300. 7957943 10.1016/0014-5793(94)01146-x

[30] Watanabe T. Katayama S. Enoki M. Honda Y. Kuwahara M. (2000) Formation of acyl radical in lipid peroxidation of linoleic acid by manganese-dependent peroxidase from *Ceriporiopsis subvermispora* and *Bjerkandera adusta*. Eur. J. Biochem. 267, 4222–4231. 10866827 10.1046/j.1432-1033.2000.01469.x

[31] Kapich A. Hofrichter M. Vares T. Hatakka A. (1999) Coupling of manganese peroxidase-mediated lipid peroxidation with destruction of nonphenolic lignin model compounds and ^14^C-labeled lignins. Biochem. Biophys, Res. Commun. 259, 212–219. 10334942 10.1006/bbrc.1999.0742

[32] Jensen K. A. Jr. Bao W. Kawai S. Srebotnik E. Hammel K. E. (1996) Manganese-dependent cleavage of nonphenolic lignin structures by *Ceriporiopsis subvermispora* in the absence of lignin peroxidase. Appl. Env. Microbiol. 62, 3679–3686. 16535418 10.1128/aem.62.10.3679-3686.1996 PMC1388956

[33] Wariishi H. Valli K. Gold M. H. (1991) *In vitro* depolymerization of lignin by manganese peroxidase of *Phanerochaete chrysosporium*. Biochem. Biophys. Res. Commun. 176, 269–275. 2018522 10.1016/0006-291x(91)90919-x

[34] Heinfling A. Ruitz-Dueñas F. J. Martinez M. J. Bergbauer M. Szewzyk U. Martinez A. T. (1998) A study on reducing substrates of manganese-oxidizing peroxidases from *Pleurotus eryngii* and *Bjerkandera adusta*. FEBS Lett. 428, 141–146. 9654123 10.1016/s0014-5793(98)00512-2

[35] Hofrichter M. (2002) Review: lignin conversion by manganese peroxidase (MnP). Enzyme Microb. Technol. 30, 454–466.

[36] Bavendamm W. (1928) Über das Vorkommen und den Nachweis von Oxydasen bei holzzerstörenden Pilzen. Zeitschr. Pflanzenkrankh. Pflanzenschtz 38, 257–276.

[37] Davidson R. W. Campbell W. A. Blaisdell D. J. (1938) Differentiation of wood decaying fungi by their reactions on gallic or tannic acid medium. J. Agr. Res. 57, 683–695.

[38] Higuchi T. (1971) Formation and biological degradation of lignins. In Adv. Enzymology (ed. Nord F. F.). vol. 34, Inter Science Publisher, New York, pp. 207–283. 10.1002/9780470122792.ch5 4947343

[39] Cameron M. D. Timofeevski S. Aust S. D. (2000) Enzymology of *Phanerochaete chrysosporium* with respect to the degradation of recalcitrant compounds and xenobiotics. Appl. Microbial Biotechnol. 54, 751–758. 10.1007/s002530000459 11152065

[40] Kawai S. Umezawa T. Higuchi T. (1988) Degradation mechanisms of phenolic ***β***-1 lignin substructure model compounds by laccase of *Coriolus versicolor.* Arch. Biochem. Biophys. 262, 99–110. 3355177 10.1016/0003-9861(88)90172-5

[41] Leontievsky A. Myasoedova N. Pozdnyakova N. Golovleva L. (1997) ‘Yellow’ laccase of *Panus tigrinus* oxidizes non-phenolic substrates without electron-transfer mediators. FEBS Lett. 413, 446–448. 9303553 10.1016/s0014-5793(97)00953-8

[42] Kirk T. K. Harkin J. M. Cowling E. B. (1968) Degradation of the lignin model compound syringylglycerol-***β***-guaiacyl ether by *Polyporus versicolor* and *Stereum frustulatum*. Biochem. Biophys. Acta 165, 145–163. 4970522 10.1016/0304-4165(68)90199-2

[43] Kawai S. Higuchi T. Nabeta K. Okuyama H. (1990) Degradation mechanisms of phenolic ***β***-O-4 lignin substructure model compounds by laccase of *Coriolus versicolor*. Biotechnology in Pulp and Paper Manufacture (eds. Kirk T. K. Chang H.-M.). Butterworth-Heinemann, USA, pp. 359–365.

[44] Bourbonnais R. Paice M. G. (1990) Oxidation of nonphenolic substrates. An expanded role for laccase in lignin biodegradation. FEBS Lett. 267, 99–102. 2365094 10.1016/0014-5793(90)80298-w

[45] Kawai S. Nakagawa M. Ohashi H. (2002) Degradation mechanisms of a nonphenolic ***β***-O-4 lignin model dimmer by *Trametes versicolor* laccase in the presence of 1-hydroxybenzotriazole. Enzyme Microb. Technol. 30, 482–489.

[46] Kawai S. Ohashi H. Hirai T. Okuyama H. Higuchi T. (1993) Degradation of syringyl lignin model polymer by laccase of *Coriolus versicolor*. Mokuzai Gakkaishi 39, 98–102.

[47] Kawai S. Umezawa T. Shimada M. Higuchi T. (1988) Aromatic ring cleavage of 4,6-di(tert-butyl)guaiacol, a phenolic lignin model compound, by laccase of *Coriolus versicolor*. FEBS Lett. 236, 309–311. 3410044 10.1016/0014-5793(88)80043-7

[48] Gierer J. Imsgard F. (1977) Studies on the autoxidation of *t*-butyl-substituted phenols in alkaline media 1. Reaction of 4-*t*-butylguaiacol. Acta Chem. Scand. B31, 537–545.

[49] Martinez A. T. (2002) Molecular biology and structure-function of lignin degrading heme peroxidases. Enzyme Microb. Technol. 30, 425–444.

[50] Akhtar M. Attridge M. C. Myers G. C. Kirk T. K. Blanchette R. A. (1992) Biomechanical pulping of loblolly pine with different strains of the white-rot fungus *Ceriporiopsis subvermispora.* TAPPI J. 75, 105–109.

[51] Kirk T. K. Blanchett R. A. Akhtar M. (1994) Biopulping: Seven Years of Consortia Research. TAPPI Proceedings 66, 57–66.

[52] Kirk T. K. (1994) Technical Overview of Forest Biotechnology Research in the U.S. TAPPI Proceedings 66, 1–4.

[53] Messner K. Srebotnik E. (1994) Biopulping: an overview of developments in an envionmentally safe paper-making technology. FEMS Microbiol. Rev. 13, 351–364.

[54] Kirk T. K. Lamar R. T. Glaser J. A. (1992) The potential of white-rot fungi in bioremediation. In Biotechnology and Environmental Science: Molecular Approaches (ed. Mongkolsuk S. et al.). Plenum Press, New York, pp. 131–138.

